# Thermolysin Versus Four Commercial Proteases in the Modification of Soy Protein Isolate: Structural, Functional, and Taste Characterization

**DOI:** 10.3390/foods15081308

**Published:** 2026-04-10

**Authors:** Xinyue Liu, Jiacheng Yin, Shuting Yin, Ping Chen, Biying Zhang

**Affiliations:** College of Food Science and Engineering, Jilin Agricultural University, Changchun 130118, Chinayst15762309862@163.com (S.Y.);

**Keywords:** protease specificity, protein hydrolysates, emulsifying properties, antioxidant activity, electronic tongue

## Abstract

The differential effects of thermolysin and four commercial proteases on soy protein isolate (SPI) were investigated under enzyme-specific hydrolysis conditions to comparatively assess the structural, functional, and instrumental taste differences among the resulting hydrolysates. Under the enzyme-specific hydrolysis conditions, among the enzymes tested, thermolysin induced substantial fragmentation of SPI, with products mainly distributed below 25 kDa and accompanied by marked conformational rearrangement. Thermolysin-treated SPI exhibited the highest total free amino acid content (14.805 mg/g), especially Tyr and Phe, together with the highest solubility (80.52 ± 4.40%), the highest emulsifying activity index (36.11 m^2^/g), and the strongest antioxidant capacities in 2,2′-azinobis(3-ethylbenzothiazoline-6-sulfonic acid) (ABTS), 2,2-diphenyl-1-picrylhydrazyl radical scavenging assay (DPPH), and hydroxyl radical scavenging assays. Electronic tongue analysis further showed that enzymatic hydrolysis generally enhanced umami and richness while reducing astringency relative to native SPI. Notably, SPI-Ther exhibited the most pronounced instrumental taste reconfiguration, characterized by increased umami (9.57) and richness (7.57), but also the highest bitterness (4.75) and aftertaste-B (3.46), indicating a distinct functionality–taste trade-off rather than simple debittering. In contrast, papain generated the highest umami response, whereas trypsin produced the mildest taste profile with the lowest bitterness. Overall, under the enzyme-specific hydrolysis conditions used in this study, thermolysin yielded the most pronounced improvement in the measured functional indices of SPI. However, these findings should be interpreted as a comparative, condition-specific assessment rather than a direct ranking of intrinsic protease specificity, and additional peptide characterization and sensory validation would be needed before taste-oriented applications can be recommended.

## 1. Introduction

Soy protein isolate (SPI) is widely used as a plant-protein ingredient because of its nutritional quality and its potential contributions to gelation, emulsification, and texture development in the modern food industry [1,2,3,4]. However, the compact globular structure of native SPI limits the accessibility of reactive groups and restricts its functional performance in some applications. Surface-directed modifications, such as complexation with 5 bioactive compounds, can improve selected properties, but they do not directly remodel the internal peptide backbone [5].

Enzymatic hydrolysis has therefore been used as a practical strategy for targeted SPI modification. Proteolytic cleavage reduces molecular size, perturbs the native conformation, and can improve hydration and interfacial behavior [6]. Hydrolysis may also be useful for the development of specialized formulations that require relatively low viscosity at high nutrient density [7]. Nevertheless, broad-spectrum hydrolysis is not always desirable. Excessive or non-selective cleavage may generate very short peptides with limited functional benefit and can intensify bitterness, which remains a major obstacle to the application of soy protein hydrolysates in foods [8,9]. Beyond Ney’s hydrophobicity rule, recent studies indicate that peptide bitterness is influenced not only by overall hydrophobicity, but also by peptide length, sequence, molecular-weight range, and the position/exposure of hydrophobic residues, because these features affect the interaction of hydrolysis products with bitter taste receptors such as TAS2Rs [10,11,12,13]. In SPI hydrolysates specifically, Dandan Liu et al. [13] further showed that relative hydrophobicity and molecular-weight distribution were among the most informative predictors of bitterness across samples prepared using different proteases and degrees of hydrolysis. In addition, many commercial proteases are less suitable for high-temperature processing, even though elevated temperature can improve reaction efficiency [14,15].

Thermolysin is a thermostable neutral metalloprotease produced by *Bacillus thermoproteolyticus* [15,16,17]. Because bitterness has often been inferred from peptide composition rather than measured directly, the effect of thermolysin on the taste profile of SPI hydrolysates remains uncertain [13,18]. Recent comparative studies have begun to examine protease-dependent SPI hydrolysis, but their scope has remained partial. For example, Li et al. [19] compared six commercial enzymes mainly in terms of DH, structural changes, functional properties, and soy milk powder performance, whereas Dandan Liu et al. [13] compared SPI hydrolysates generated by different enzymes and DH levels primarily to model bitterness. However, a side-by-side comparison that includes thermolysin and simultaneously links enzyme-dependent structural remodeling, functional performance, antioxidant response, free amino acid release, and instrumental taste profile under practical enzyme-specific hydrolysis conditions remains limited. In the present study, thermolysin was compared with four commercial proteases—alkaline protease, papain, neutral protease, and trypsin—to comparatively evaluate protease-dependent differences in the structural characteristics, functional properties, and instrumental taste profile of SPI hydrolysates under practical, enzyme-specific hydrolysis conditions. A limitation of the present study is that each enzyme was evaluated under its own literature-supported operating pH and temperature rather than under a unified reaction environment. Therefore, the observed differences should be interpreted as the combined effects of protease type and reaction conditions, rather than as a direct comparison of intrinsic protease specificity alone. Microstructure, molecular-weight distribution, secondary structure, free amino acids, solubility, emulsifying properties, antioxidant activity, and electronic tongue responses were evaluated to provide a comparative assessment of thermolysin-mediated SPI hydrolysis and to explore whether functional enhancement was accompanied by an instrumental sensory trade-off.

## 2. Materials and Methods

### 2.1. Preparation of SPI Hydrolysates

A 4% (*w*/*v*) soy protein isolate (SPI) solution was prepared by dispersing SPI (protein content, 92.5 g/100 g; Shandong Wonderful Biotechnology Co., Ltd., Dongying, China) in ultrapure water according to Xia et al. [20]. Hydrolysis was carried out at an enzyme-to-substrate ratio of 1:50 (*w*/*w*) under continuous stirring at 300 rpm. The present design was intended to compare practical enzyme treatments rather than to isolate intrinsic protease specificity under a unified reaction system. Similarly to Li et al. [19], this study also applied enzyme-specific temperature and pH conditions within the respective applicable operating ranges for each protease. Accordingly, the differences observed among hydrolysates in this study should be interpreted as the combined effects of protease type and process conditions, rather than as a direct comparison of enzyme specificity alone. For thermolysin, hydrolysis was conducted at 70 °C according to Di Bernardini et al. [21]. The conditions used for alkaline protease, papain, neutral protease, and trypsin were adapted from Meinlschmidt et al. [22], Fan et al. [23], Li et al. [19] and Lee [24], respectively. The pH was continuously monitored using a pH meter (PB-10, Sartorius, Shanghai, China) and maintained constant by adding 0.1 M HCl (Beijing Solarbio Science & Technology Co., Ltd., Beijing, China) or 0.1 M NaOH (Beijing Solarbio Science & Technology Co., Ltd., Beijing, China) [25]. In addition, Meinlschmidt et al. [26] showed that, for SPI, substantial hydrolysis had already occurred within 60 min, with the degree of hydrolysis reaching 10.1% for Alcalase and 7.2% for Corolase 2TS at that time point. Similarly, Fan et al. [23] reported that SPI hydrolyzed for 60 min reached a DH of 11.23% with alkaline protease and 1.3% with papain, while the hydrolysis rate gradually leveled off with further extension of time. Moreover, Dent et al. [27] found that prolonging hydrolysis continued to alter soy protein properties and could even reduce solubility because of structural changes and aggregate formation. Therefore, a fixed hydrolysis time of 1 h was used in this study as a literature-supported limited hydrolysis window for comparison among enzyme treatments, allowing measurable modification while limiting the additional complexity associated with more extensive hydrolysis. After hydrolysis, the enzymes were inactivated in a 90 °C water bath for 10 min [28]. After cooling to room temperature, the hydrolysates were neutralized and centrifuged using a refrigerated centrifuge (TGL-20000CR, Shanghai Anting Scientific Instrument Factory, Shanghai, China) at 10,000× *g* for 15 min at 4 °C [29]. The supernatants were then collected and lyophilized using a freeze dryer (LGJ-12, Beijing Songyuan Huaxing Technology Development Co., Ltd., Beijing, China; cold-trap temperature ≤−60 °C and ultimate vacuum ≤1 Pa, according to the manufacturer’s specifications) to obtain the hydrolysate powders. All samples were freeze-dried under the same instrument settings and operating procedure to ensure comparability among treatments.

### 2.2. Scanning Electron Microscopy (SEM)

The surface morphology of native SPI and its enzymatic hydrolysates was characterized using a scanning electron microscope (SU8600, Hitachi, Tokyo, Japan). The hydrolysate powders obtained after lyophilization were sputter-coated with gold before observation. SEM images were recorded at an accelerating voltage of 3.00 kV. For each sample, representative micrographs were acquired at two magnification levels (×50, ×200) to assess both the overall surface architecture and finer local features.

### 2.3. Sodium Dodecyl Sulfate Polyacrylamide Gel Electrophoresis (SDS-PAGE)

Based on the protein concentration results shown in Table 1, aliquots containing 30 μg of protein were prepared. The aliquots were mixed with NuPAGE LDS Sample Buffer (4×; Shanghai Yuanye Bio-Technology Co., Ltd., Shanghai, China), heated in a boiling water bath for 10 min for denaturation, and then centrifuged at 14,000× *g* for 5 min. The resulting supernatants were separated by SDS-PAGE on a 15% polyacrylamide gel using a standard Tris-glycine running buffer (25 mM Tris, 192 mM glycine, 0.1% SDS, pH 8.3). Electrophoresis was performed using a DYY-6B electrophoresis apparatus (Beijing Liuyi Instrument Factory, Beijing, China) at a constant voltage of 120 V for 60 min. A pre-stained protein marker (BF102, Beijing Bangfei Bioscience Co., Ltd., Beijing, China) was loaded alongside the samples as a molecular weight marker. After electrophoresis, the gels were stained with Coomassie Brilliant Blue (Shanghai Yuanye Bio-Technology Co., Ltd., Shanghai, China) and destained until the background became clear. Gel images were recorded using a chemiluminescence imaging system (Tanon 4200, Tanon Science & Technology Co., Ltd., Shanghai, China).

### 2.4. Fourier Transform Infrared Spectroscopy

Fourier transform infrared spectroscopy (FTIR) spectra of all samples were recorded according to Yin et al. [30], with minor modifications, by using a Spectrum 100 FTIR spectrometer (PerkinElmer, Waltham, MA, USA) equipped with an ATR accessory. Before measurement, a background spectrum of air was recorded. The lyophilized powder was placed directly onto the crystal cell and secured with consistent pressure to ensure good contact. Spectra were collected in the range of 4000–600 cm^−1^ with a resolution of 4 cm^−1^. For each sample, 16 scans were co-added and averaged to improve the signal-to-noise ratio and ensure reproducibility.

### 2.5. Circular Dichroism (CD) Spectroscopy

CD spectra were recorded according to Zang et al. [31] with minor modifications. Freeze-dried samples were dissolved in 10 mM phosphate buffer (pH 7.0), prepared from NaH_2_PO_4_·2H_2_O and Na_2_HPO_4_·12H_2_O (Beijing Solarbio Science & Technology Co., Ltd., Beijing, China), to a final concentration of 0.2 mg/mL. For each treatment, three independently prepared hydrolysate batches were combined in equal proportions to obtain one composite sample for CD measurement. The secondary structural changes in the enzymatic hydrolysates were then analyzed using a circular dichroism spectropolarimeter (J-1500, JASCO, Tokyo, Japan). Prior to measurement, the optical system was purged with high-purity nitrogen for 15 min, and a constant nitrogen flow was maintained throughout the experiment. An aliquot of 400 μL of each sample solution was transferred into a quartz cuvette with a path length of 0.1 cm. Spectra were recorded over the range of 190–260 nm at 25 °C with a scanning speed of 100 nm/min and a bandwidth of 1.0 nm. Three parallel measurements were performed for each composite sample, and the corresponding spectra were averaged to improve the signal-to-noise ratio. The corresponding buffer spectrum was subtracted as the background. The relative contents of α-helix, β-sheet, β-turn, and random coil were estimated using the CDPro software (version 2021) package.

### 2.6. Analysis of Free Amino Acids in Enzymatic Hydrolysates

The free amino acid composition of different protein hydrolysates was analyzed with modifications to the method described by Yin et al. [32]. Briefly, for each treatment, three independently prepared hydrolysate batches were combined in equal proportions to obtain one composite sample. Then 1.0 g of sample was accurately weighed into a 10 mL volumetric flask and dissolved in ultrapure water to volume. After complete dissolution, the solution was kept at 4 °C for 24 h. To remove unhydrolyzed macromolecular proteins, the supernatant was mixed with an equal volume of 5% sulfosalicylic acid solution (Sigma-Aldrich, St. Louis, MO, USA) and allowed to stand at 4 °C for 2 h to ensure complete precipitation. The mixture was then centrifuged at 10,000× *g* for 15 min at 4 °C, and the resulting supernatant was filtered through a 0.22 μm aqueous-phase membrane filter (Thermo Fisher Scientific, Waltham, MA, USA) to obtain the test solution. Amino acid analysis was performed using an automatic amino acid analyzer (L-8900, Hitachi, Japan) equipped with a cation exchange column. Sodium citrate-citric acid buffers with pH values of 3.2, 3.3, 4.0, and 4.9 and a 4% ninhydrin buffer solution were obtained from Beijing Solarbio Science & Technology Co., Ltd. (Beijing, China). Amino acids were identified and quantified by comparing the retention times and peak areas of the samples with those of a standard amino acid mixture (Sigma-Aldrich, St. Louis, MO, USA). For each composite sample, three parallel injections were performed on the automatic amino acid analyzer.

### 2.7. Determination of Solubility

According to Sui et al. [33], protein samples were dispersed in phosphate-buffered saline (PBS, pH 7.0) at a concentration of 3 mg/mL. To ensure comparability with the emulsification measurements, solubility was determined under this fixed neutral condition. The mixture was stirred continuously at room temperature for 30 min to ensure adequate hydration and was then centrifuged at 10,000× *g* for 20 min at 4 °C using a refrigerated centrifuge (TGL-20000CR, Shanghai Anting Scientific Instrument Factory, Shanghai, China). The soluble protein concentration in the resulting supernatant was quantified using a BCA protein assay kit (Biosharp, Beijing, China).

### 2.8. Determination of Emulsifying Activity and Stability

The emulsifying properties were evaluated according to the method described by Sui et al. [33], with minor modifications. Briefly, SPI and its enzymatic hydrolysate samples were dispersed in phosphate-buffered saline (PBS, 10 mM, pH 7.0), so that emulsifying activity and stability were measured under the same neutral condition used for the solubility assay. The protein dispersions were mixed with soybean oil (Arawana, Yihai Kerry Arawana Holdings Co., Ltd., Shanghai, China) at a ratio of 3:1 (*v*/*v*), and the mixtures were homogenized at 20,000 rpm for 1 min using a high-speed homogenizer (HX-4GM, Shanghai Huxi Industrial Co., Ltd., Shanghai, China). Aliquots of the freshly prepared emulsions were immediately diluted in 1% (*w*/*v*) sodium dodecyl sulfate (SDS) solution (Sigma, USA). The absorbance of the diluted emulsions was measured at 500 nm using a UV-Vis spectrophotometer (UV-7200, Unico, Shanghai, China) immediately after homogenization (0 min) and again after 10 min. The emulsifying activity index (EAI) and emulsion stability index (ESI) were calculated using the following equations:

$$EAI\left(m^{2}/g\right)=\frac{2\times 2.303\times A_{0}\times DF}{10,000\times \theta \times L\times C}$$

$$ESI\left(min\right)=\frac{A_{0}\times 10}{A_{0}-A_{10}}$$

where A_0_ and A_10_ are the absorbance values measured at 0 min and 10 min, respectively; DF is the dilution factor (typically 100); θ is the volume fraction of the oil phase in the emulsion (0.25); L is the optical path length of the cuvette (1 cm); C is the initial protein concentration (g/mL); and 10,000 is the conversion factor used to express the result in m^2^/g.

### 2.9. ABTS Radical Scavenging Activity

ABTS radical scavenging activity was determined with modifications to the method of Pi et al. [34]. The sample concentration was adjusted to 1.0 mg/mL. Then, 50 μL of sample solution was mixed with 150 μL of ABTS working solution (Sigma-Aldrich, St. Louis, MO, USA) and reacted in the dark at room temperature for 6 min. The absorbance at 734 nm was measured using a microplate reader (SpectraMax 190, Molecular Devices, San Jose, CA, USA). To correct for sample interference, a mixture of 50 μL of sample and 150 μL of distilled water was used as the blank, and a mixture of distilled water and ABTS solution without sample was used as the control. The scavenging activity was calculated using the following equation:

$$ABTS\;Scavenging\;Activity\left(\%\right)=(1-\frac{A_{sample}-A_{blank}}{A_{0}})\times 100$$

### 2.10. DPPH Radical Scavenging Activity

DPPH radical scavenging activity was determined with modifications to the method of Pi et al. [34]. Briefly, 100 μL of sample solution (1.0 mg/mL) was mixed with 100 μL of 0.2 mM DPPH solution (Beijing Solarbio Science & Technology Co., Ltd., Beijing, China) prepared in absolute ethanol (Beijing Solarbio Science & Technology Co., Ltd., Beijing, China) and incubated in the dark at room temperature for 30 min. The absorbance at 517 nm was measured using a microplate reader (SpectraMax 190, Molecular Devices, USA). A mixture of sample and ethanol without DPPH was used as the blank, and a mixture of ethanol and DPPH solution without sample served as the control. The scavenging activity was calculated using the following equation.

$$DPPH\;radical\;scavenging\;activity\left(\%\right)=(1-\frac{A_{sample}{-A}_{blank}}{A_{0}})\times 100$$

### 2.11. Hydroxyl Radical Scavenging Activity

Hydroxyl radical scavenging activity was determined by the salicylic acid method with minor modifications to the procedure of Ren et al. [35]. Briefly, 50 μL of sample solution (2.0–10.0 mg/mL) was mixed with FeSO_4_ and H_2_O_2_ solutions to initiate the Fenton reaction (Sigma-Aldrich, St. Louis, MO, USA), followed by the addition of salicylic acid (Sigma-Aldrich, St. Louis, MO, USA). After incubation at 37 °C for 60 min in the dark, the absorbance was measured at 510 nm using a microplate reader (SpectraMax 190, Molecular Devices, USA). Distilled water was used instead of the sample for the control group (A_0_), while the blank group (A_c_) was prepared without H_2_O_2_ to correct for background interference. The scavenging activity was calculated using the following equation.

$$\;\mathrm{Hydroxyl}\;\mathrm{radical}\;\mathrm{scavenging}\;\mathrm{ratio}=(A_{s}-A_{c})\;/\;(A_{0}-A_{c})$$

where A_S_ represents the absorbance of the sample group.

### 2.12. Electronic Tongue (e-Tongue)

The taste profiles of native SPI and its enzymatic hydrolysates (SPI-Ther, SPI-Alk, SPI-Pap, SPI-Neu, and SPI-Try) were evaluated using an SA402B electronic tongue system (Insent Inc., Atsugi, Japan) according to Du et al. [36] and Wang et al. [37], with minor modifications. The system was equipped with an array of artificial lipid membrane sensors and corresponding reference electrodes for the evaluation of sourness, saltiness, astringency, umami, richness, bitterness, sweetness, and aftertaste-related attributes. For each treatment, three independently prepared hydrolysate batches were combined in equal proportions to obtain one composite sample for electronic tongue analysis. For sample preparation, 1.0 g of each lyophilized sample was dissolved in deionized water, homogenized, centrifuged at 4000× *g* for 10 min, and filtered through a 0.45 μm membrane. Before measurement, the sensors were conditioned and checked according to the standard SA402B operating procedure until stable baseline responses were obtained (baseline drift < 0.5 mV). The reference solution consisted of 30 mM KCl and 0.3 mM tartaric acid. During analysis, the sensor potentials in the reference solution were first recorded, after which the sensor array was immersed in each sample filtrate for 30 s. Taste intensity was calculated from the potentiometric difference between the sample solution and the reference solution. After sample measurement, the sensors were transferred to the CPA (change in membrane potential caused by adsorption) solution for 30 s to determine aftertaste-related attributes. Each composite sample was measured in triplicate.

### 2.13. Statistical Analysis

Except for CD spectroscopy, free amino acid analysis, and electronic tongue analysis, all measurements were performed using three independently prepared hydrolysate batches, and the results are expressed as mean ± standard deviation (SD). For CD spectroscopy, free amino acid analysis, and electronic tongue analysis, three independently prepared hydrolysate batches were first combined in equal proportions to obtain one composite sample for each treatment, and the composite sample was then analyzed in triplicate. Therefore, the replicate values obtained for these three analyses reflect parallel measurements of pooled samples rather than variation among independent hydrolysate batches. Statistical analysis was performed using SPSS Statistics 27.0 (IBM, Armonk, NY, USA). Normality and homogeneity of variance were assessed by the Shapiro–Wilk test and Levene’s test, respectively. Since these assumptions were satisfied, differences among groups were analyzed by one-way analysis of variance (ANOVA) followed by Duncan’s multiple range test. Differences were considered statistically significant at *p* < 0.05. Figures were prepared using Origin 2024.

## 3. Results

### 3.1. SEM

**Figure 1:**
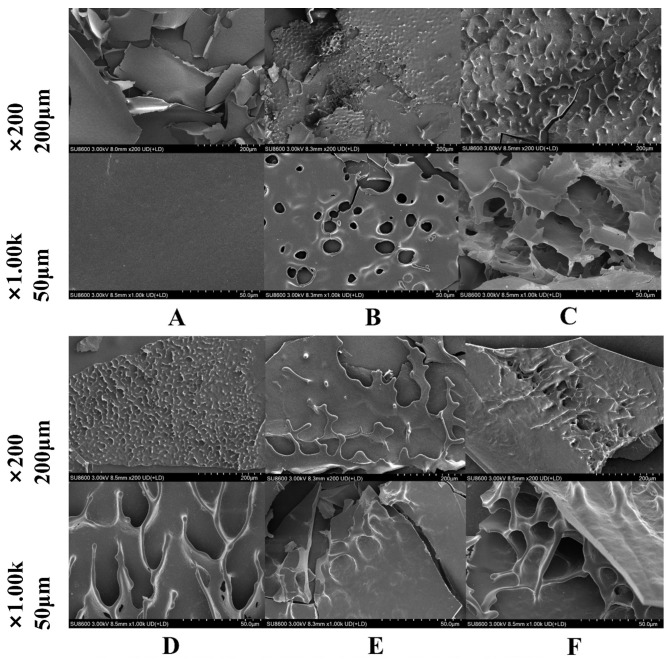
Surface morphology of SPI and SPI hydrolysates prepared by five different proteases. Note: (**A**): SPI; (**B**): SPI-Ther; (**C**): SPI-Alk; (**D**): SPI-Pap; (**E**): SPL-Try; (**F**): SPI-Neu.

Figure 1 shows clear protease-dependent differences in the SEM morphology of native SPI and its hydrolysates. Native SPI (Figure 1A) presented a compact lamellar structure with a relatively smooth and continuous surface. After enzymatic hydrolysis, this dense morphology was disrupted to different extents. SPI-Ther (Figure 1B) developed a rough and perforated surface with numerous circular pores, whereas SPI-Alk (Figure 1C) showed more extensive fragmentation, collapsed lamellar regions, and irregular cavities. SPI-Pap (Figure 1D) displayed a wrinkled and partially opened structure with elongated voids, while SPI-Try (Figure 1E) largely retained a more intact sheet-like morphology, showing only limited cracking. SPI-Neu (Figure 1F) showed a looser and more open porous network than native SPI.

These observations indicate that enzymatic hydrolysis disrupted the original compact SPI structure to different extents and generated distinct surface architectures depending on protease type. Similar enzyme-dependent structural remodeling of SPI, accompanied by changes in microstructure and downstream functional behavior, has been reported in recent studies of selective and differential enzymatic hydrolysis of soy proteins [19,23]. Because the present SEM images were obtained from lyophilized powders, they should be interpreted comparatively rather than as artifact-free representations of the hydrated native state. Nevertheless, the more open and disrupted morphologies observed here are consistent with the subsequent differences in solubility, emulsifying behavior, and antioxidant performance.

### 3.2. SDS-PAGE

**Figure 2:**
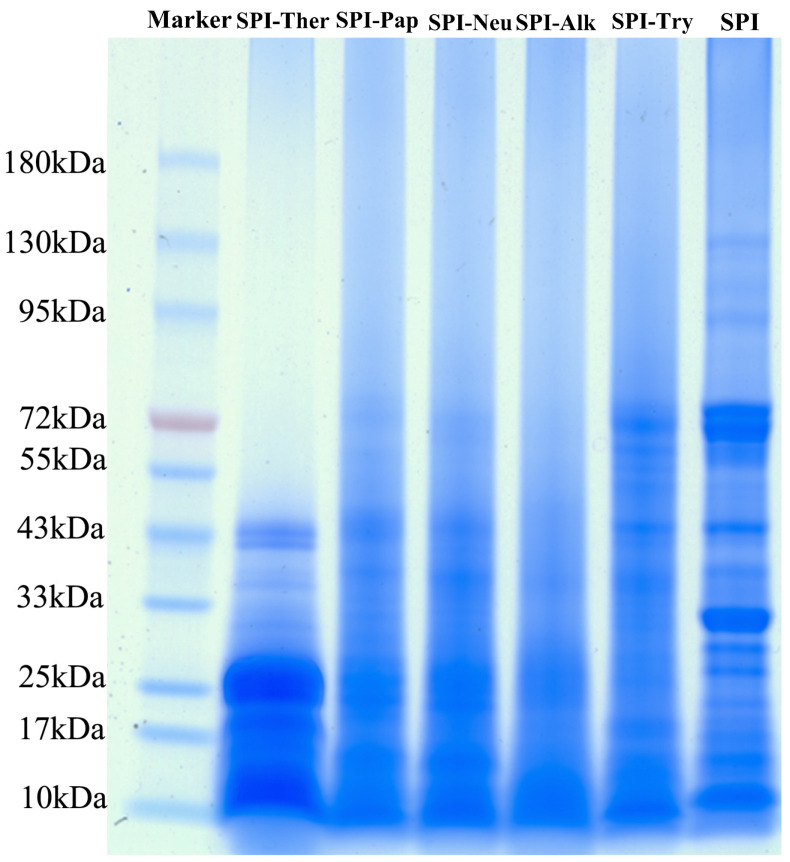
SDS-PAGE profiles of soy protein isolate (SPI) and its enzymatic hydrolysates.

SDS-PAGE (Figure 2) showed a general shift in protein bands toward lower molecular weight regions after enzymatic hydrolysis, indicating that the extent of protein breakdown differed among proteases under the tested conditions. The most extensive disappearance of high-molecular-weight bands was observed for SPI-Alk, followed by SPI-Ther, whereas SPI-Try retained more bands in the higher molecular weight region, which is consistent with previous reports on the strong hydrolytic efficiency of alkaline protease toward SPI. SPI-Ther also showed substantial fragmentation, with most bands distributed below 25 kDa, which is consistent with the cleavage preference of thermolysin for peptide bonds adjacent to bulky hydrophobic residues and its effective hydrolytic performance under elevated-temperature conditions [21].

By comparison, SPI-Pap and SPI-Neu showed intermediate hydrolysis patterns, whereas SPI-Try displayed the lowest apparent degree of degradation. The relatively limited degradation observed for SPI-Try may be associated with the strict specificity of trypsin for cleavage at the carboxyl side of lysine and arginine residues and the restricted accessibility of these sites in folded soy protein structures. Because no densitometric analysis, degree-of-hydrolysis determination, or chromatographic peptide-size profiling was performed in the present study, the gel patterns should be interpreted only as qualitative evidence of differential fragmentation rather than as a quantitative measure of hydrolysis intensity. Therefore, any relationship between the SDS-PAGE patterns and the subsequent functional or taste results is discussed here as a qualitative tendency only, and quantitative hydrolysis indices should be included in future work. Overall, the SDS-PAGE results are interpreted here primarily as qualitative molecular-size evidence of protease-dependent fragmentation among SPI hydrolysates.

### 3.3. FTIR

**Figure 3:**
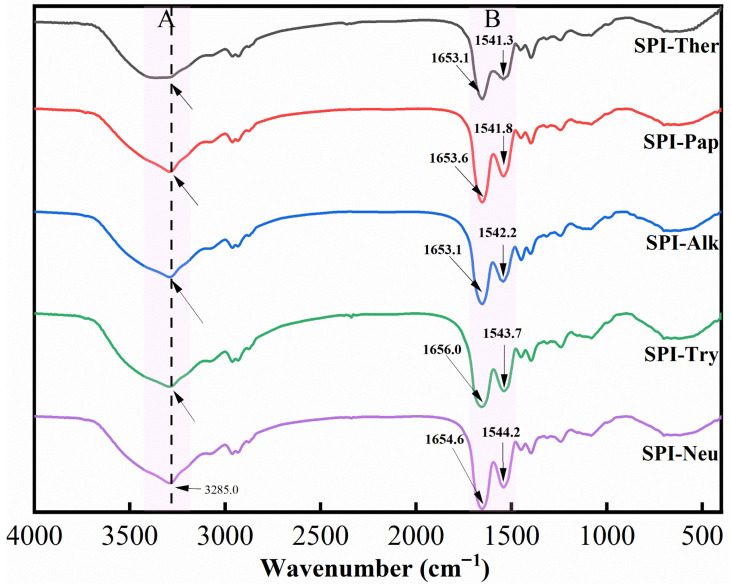
FTIR spectra of soy protein isolate (SPI) hydrolysates prepared by different proteases.

As shown in Figure 3, the FTIR spectra exhibited clear protease-dependent differences among the SPI hydrolysates, indicating that enzymatic hydrolysis altered the molecular environment of the protein backbone. This overall pattern is broadly consistent with previous studies showing that limited hydrolysis of SPI changes the amide-band profile through alterations in hydrogen bonding and backbone conformation [19,38]. Variations were observed mainly in the amide A, amide I, and amide II regions. In the amide A region (around 3285 cm^−1^), SPI-Ther displayed a broader and less distinct absorption profile than the other groups, suggesting that thermolysin treatment induced a different hydrogen-bonding environment after hydrolysis.

In the amide I region (around 1654 cm^−1^), spectral differences were observed among the hydrolysates, indicating protease-dependent changes in the backbone conformational environment. In the amide II region (around 1541 cm^−1^), SPI-Ther, SPI-Alk, and SPI-Pap also showed more obvious spectral variation than SPI-Try and SPI-Neu, further supporting qualitative differences in the amide-band environment and structural rearrangement among protease treatments. Overall, the FTIR results indicate that thermolysin and alkaline protease induced stronger changes in the amide-band environment of SPI under the tested conditions. In the present study, FTIR is interpreted primarily as qualitative evidence of backbone and hydrogen-bonding rearrangement, whereas SDS-PAGE provides molecular-size information and CD provides quantitative estimates of secondary-structure redistribution.

### 3.4. CD

**Figure 4:**
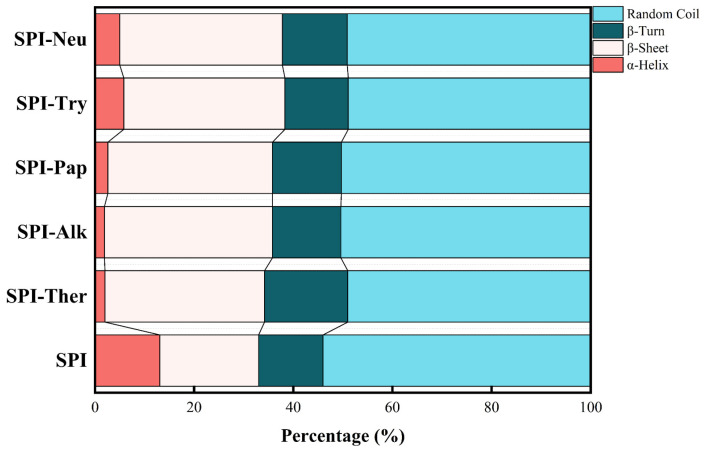
Secondary structure composition of soy protein isolate (SPI) and its enzymatic hydrolysates.

As showed as Table 2 and Figure 4, CD spectroscopy showed that enzymatic hydrolysis induced pronounced, protease-dependent changes in the secondary structure of SPI. Compared with native SPI, all hydrolysates exhibited decreased α-helix content and increased β-sheet content to varying extents, indicating substantial conformational rearrangement after proteolysis. This trend is generally consistent with recent reports showing that enzymatic hydrolysis of SPI reduces ordered secondary structures and promotes structural redistribution in a protease-dependent manner [19]. As shown in Table 2, compared with native SPI, all hydrolysates exhibited decreased α-helix content and increased β-sheet content to varying extents, indicating substantial conformational rearrangement after proteolysis. Among the treatments, SPI-Alk showed the most pronounced structural transition, with α-helix content decreasing to 1.9% and β-sheet content increasing to 33.9%. This result indicates that alkaline protease treatment caused the greatest redistribution of secondary-structure elements under the tested conditions [39], thereby increasing the accessibility of cleavage sites and enabling efficient hydrolysis by the broad-specificity alkaline protease [40].

SPI-Ther also underwent substantial conformational rearrangement, with α-helix content decreasing from 13.1% to 2.0% and β-sheet content increasing to 32.2%. This finding likewise supports marked secondary-structure reorganization after thermolysin hydrolysis [19,41,42]. By comparison, SPI-Pap, SPI-Try, and SPI-Neu retained relatively higher α-helix contents, suggesting milder secondary-structure perturbation under the tested conditions. Overall, the CD results suggest protease-dependent redistribution of secondary-structure elements after hydrolysis, complementing the FTIR observations and the qualitative molecular-size evidence obtained by SDS-PAGE.

### 3.5. Free Amino Acids

**Figure 5:**
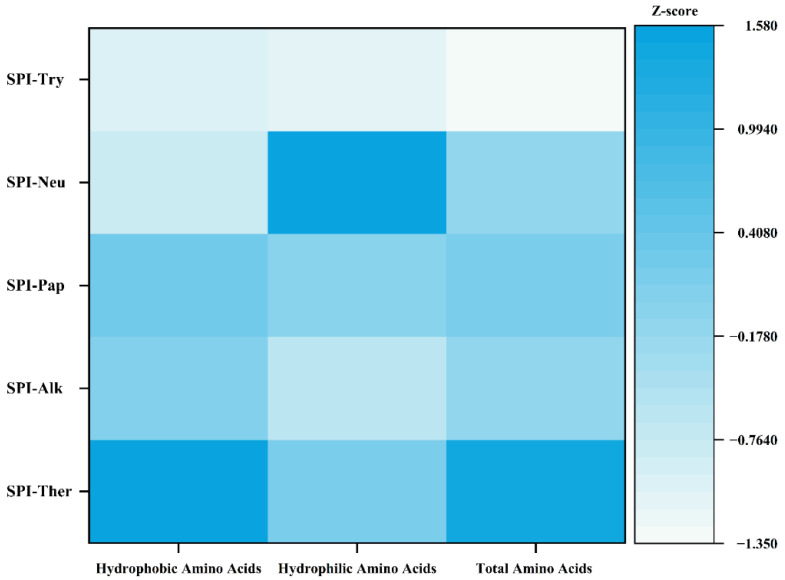
Heat map analysis of free amino acid profiles in soy protein isolate (SPI) hydrolysates prepared by different proteases. The color scale represents the Z-score standardization of amino acid contents, where darker blue indicates higher relative content and lighter color indicates lower content.

The Z-score heatmap in Figure 5 and the quantitative data in Table 3 showed clear differences in free amino acid release among the protease treatments. These protease-dependent differences in total free amino acid release are broadly consistent with previous comparative studies on SPI hydrolysis, in which different commercial enzymes generated distinct amino acid release patterns and markedly different total FAA levels [19]. SPI-Ther exhibited the highest total free amino acid content (14.805 mg/g), whereas SPI-Neu showed the lowest value (5.507 mg/g), indicating that thermolysin released more free amino acids under the tested conditions. The amino acid distribution also differed among proteases. In SPI-Ther, Tyr and Phe increased markedly and reached 4.928 mg/g and 3.355 mg/g, respectively, which is consistent with the known cleavage preference of thermolysin for peptide bonds associated with hydrophobic residues [43]. In contrast, SPI-Try showed the highest Arg content (2.888 mg/g), consistent with the substrate specificity of trypsin for lysine- and arginine-containing cleavage sites [44]. SPI-Pap and SPI-Alk showed more balanced amino acid distributions, whereas SPI-Neu retained the lowest overall level of free amino acid release.

These results indicate that different proteases generated distinct amino acid release patterns and hydrolysis outcomes, but the compositional differences should be interpreted primarily as evidence of differential proteolysis rather than as direct proof of superior functional performance. Likewise, the present free amino acid data should not be used alone to predict bitterness or other taste attributes, because sensory and functional responses are also likely to be influenced substantially by peptide-related factors, including peptide sequence, chain length, and molecular distribution, which were not directly characterized in the present study.

### 3.6. Solubility

**Figure 6:**
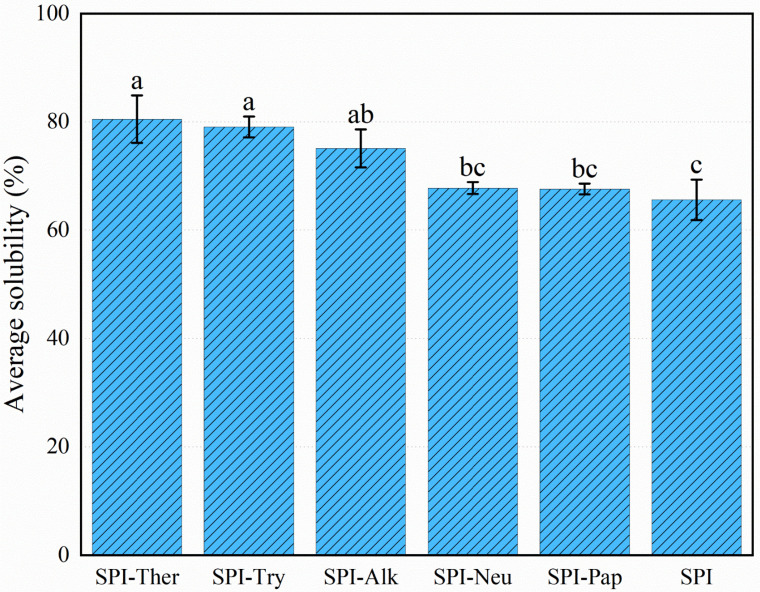
Solubility of soy protein isolate (SPI) and its hydrolysates prepared by different proteases. Note: Values are expressed as mean ± SD (n = 3). Different lowercase letters above the bars indicate significant differences (*p* < 0.05).

All enzyme treatments altered SPI solubility (Figure 6). Native SPI showed a solubility of 65.62 ± 3.74%. The highest value was observed for SPI-Ther (80.52 ± 4.40%), followed by SPI-Try (79.09 ± 1.93%). SPI-Alk also showed higher solubility than native SPI, but the increase was smaller than those of SPI-Ther and SPI-Try. Although the exact ranking differed from some previous reports, the general improvement in solubility after enzymatic hydrolysis is consistent with earlier studies on SPI, which showed that limited hydrolysis can enhance solubility by reducing molecular size and exposing hydrophilic groups [19,25]. SPI-Neu and SPI-Pap showed only limited improvement relative to native SPI. The improved solubility of SPI-Ther may be related to the combined effects of reduced molecular size and conformational rearrangement, which could enhance protein–water interactions [45]. Because soy protein solubility is highly pH-dependent, the present values were obtained only in the tested PBS system at pH 7.0 and should be interpreted as condition-specific results rather than as universal behavior across all food matrices or pH environments.

### 3.7. Emulsifying Activity and Stability

**Figure 7:**
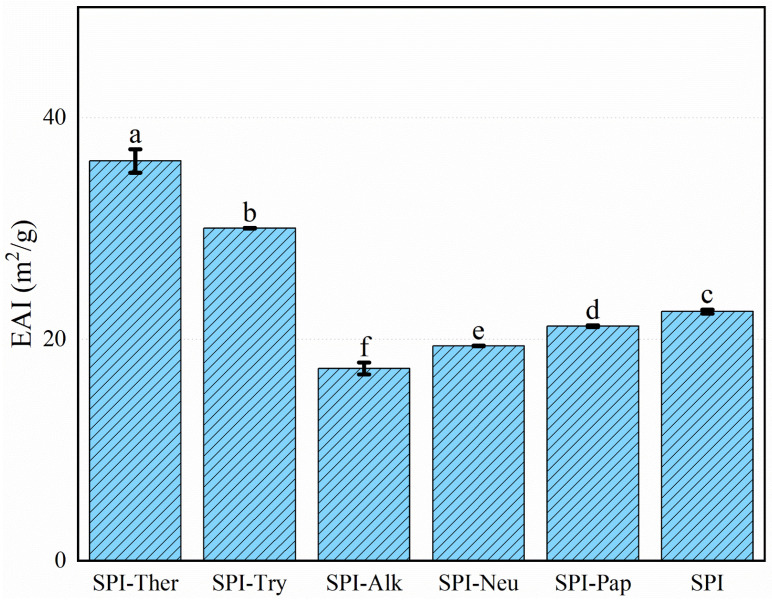
Emulsifying activity index (EAI) of soy protein isolate (SPI) and its hydrolysates prepared by different proteases. Different lowercase letters above the bars indicate significant differences (*p* < 0.05).

**Figure 8:**
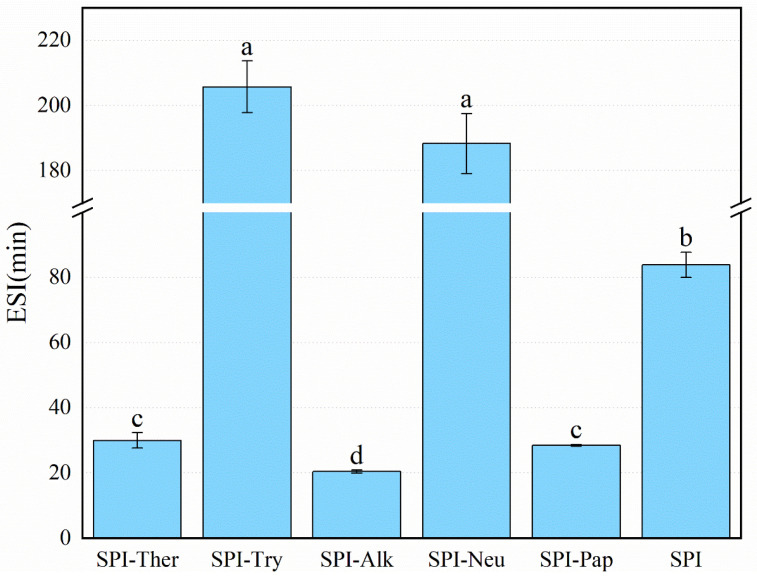
Emulsifying stability index (ESI) of soy protein isolate (SPI) and its hydrolysates prepared by different proteases. Different lowercase letters above the bars indicate significant differences (*p* < 0.05).

Emulsifying activity and stability differed significantly among the treatments (Figure 7 and Figure 8). SPI-Ther showed the highest emulsifying activity index (EAI, 36.11 m^2^/g), followed by SPI-Try (30.04 m^2^/g). The lowest EAI was observed for SPI-Alk (17.37 m^2^/g). The particularly low EAI of SPI-Alk may reflect excessive fragmentation under the tested alkaline hydrolysis conditions. This interpretation is consistent with reports that broad-specificity proteases may improve interfacial activity only up to a certain hydrolysis extent, beyond which emulsion stability and, in some cases, EAI may decline [46,47]. In contrast, the highest emulsion stability index (ESI) values were obtained for SPI-Try (205.75 min) and SPI-Neu (188.30 min), whereas SPI-Ther and SPI-Pap showed lower stability. These results indicate that the structural features favoring rapid adsorption at the oil–water interface were not necessarily the same as those favoring long-term interfacial film stability. This distinction between EAI and ESI is broadly consistent with the literature, which shows that the structural features promoting rapid interfacial adsorption are not necessarily those that favor long-term film stability. Previous studies on soy protein hydrolysates likewise reported that moderate hydrolysis may enhance emulsifying activity, whereas more extensive fragmentation can weaken interfacial cohesion and reduce emulsion stability [19,25,46]. The high EAI of SPI-Ther is consistent with its improved solubility and marked conformational change, whereas the high ESI of SPI-Try may suggest that the peptides generated by trypsin formed a more cohesive interfacial film [45,46]. Extensive hydrolysis by alkaline protease was unfavorable for both EAI and ESI [48]. However, all EAI and ESI measurements in the present study were conducted in a PBS/oil system at pH 7.0, and therefore the observed rankings should be interpreted as applying to this tested neutral condition rather than being directly generalized to other pH conditions or food formulation environments without further validation.

### 3.8. ABTS Radical Scavenging Activity

**Figure 9:**
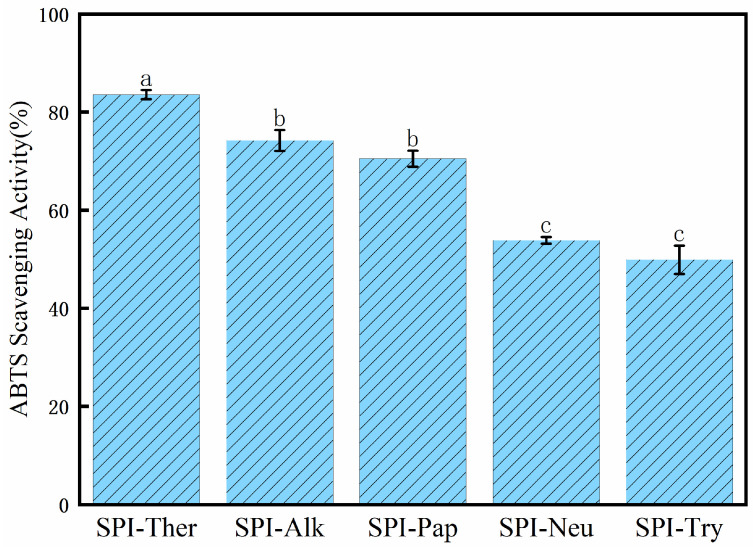
ABTS radical scavenging activity of soy protein isolate (SPI) hydrolysates prepared with different proteases. Different lowercase letters above the bars indicate significant differences (*p* < 0.05).

As shown in Figure 9, SPI-Ther showed the highest ABTS radical scavenging activity, followed by SPI-Alk and SPI-Pap. Combined with the DPPH and hydroxyl radical scavenging results, these findings indicate that thermolysin treatment produced the most pronounced overall improvement in the in vitro chemical radical-scavenging properties of SPI under the tested conditions. The enhanced activity may be associated with thermolysin-induced structural modification, including reduced molecular size, altered peptide distribution, and increased exposure of residues capable of participating in radical quenching. Similar trends have been reported for protein hydrolysates in which enzymatic cleavage improved antioxidant performance through changes in molecular conformation and peptide composition [49,50]. Overall, the present results suggest that the antioxidant response of SPI hydrolysates was strongly dependent on protease type and the resulting structural characteristics. However, these ABTS results should be interpreted only as evidence of relative antioxidant potential in the tested in vitro system, rather than as direct evidence of biological or physiological antioxidant efficacy. The strong protease dependence observed in the ABTS assay is broadly consistent with previous studies on SPI hydrolysates, where different enzymes generated markedly different in vitro antioxidant responses depending on peptide size distribution and exposed redox-active residues [19,49].

### 3.9. DPPH Radical Scavenging Activity

**Figure 10:**
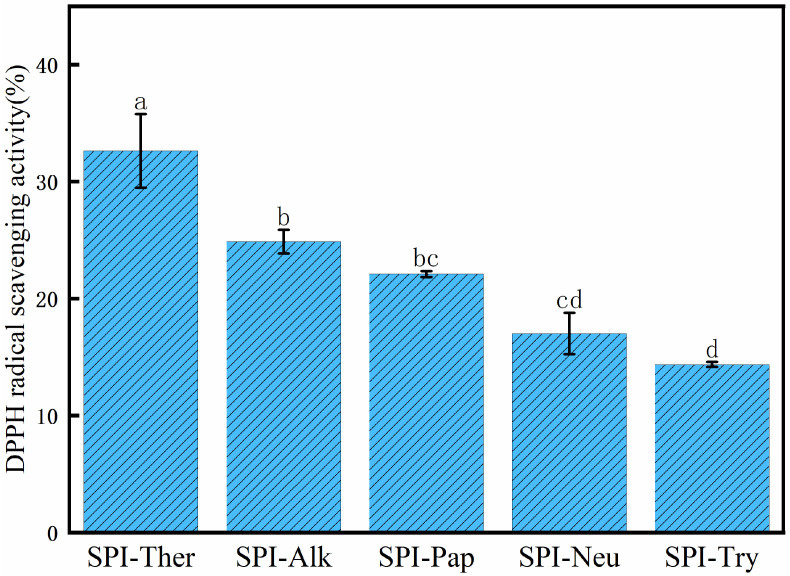
DPPH radical scavenging activity of soy protein isolate (SPI) hydrolysates prepared with different proteases. Different lowercase letters above the bars indicate significant differences (*p* < 0.05).

A similar ranking was observed in the DPPH assay (Figure 10). SPI-Ther showed the strongest DPPH radical scavenging activity (approximately 33%), followed by SPI-Alk and SPI-Pap, whereas SPI-Neu and SPI-Try showed lower activity. The agreement between the ABTS and DPPH assays indicates that thermolysin treatment improved the antioxidant properties of SPI under the tested conditions. Nevertheless, the results should still be interpreted as in vitro chemical indicators of antioxidant potential rather than as evidence of physiological efficacy [49]. In particular, the DPPH assay reflects radical-quenching behavior in a simplified chemical system and does not necessarily predict antioxidant performance in vivo. A similar enzyme-dependent ranking has also been reported in previous studies of SPI hydrolysates, in which broad-specificity or more efficient hydrolysis systems often yielded stronger DPPH scavenging capacity than milder treatments [49].

### 3.10. Hydroxyl Radical Scavenging Activity

**Figure 11:**
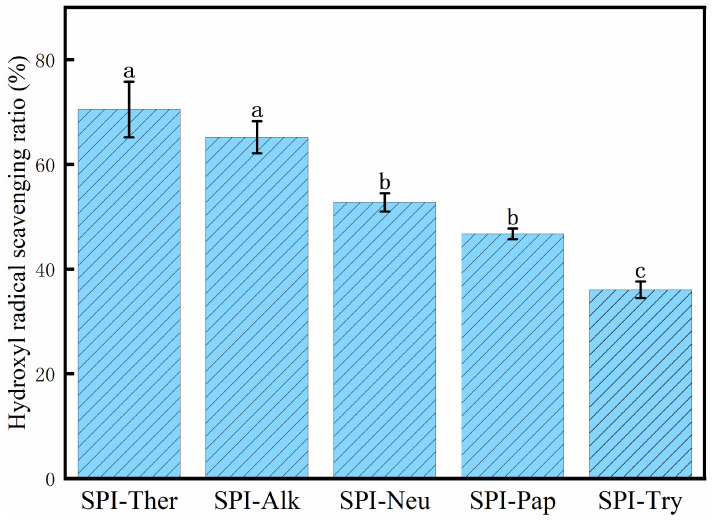
Hydroxyl radical scavenging ratio of soy protein isolate (SPI) hydrolysates prepared by different proteases. Different lowercase letters above the bars indicate significant differences (*p* < 0.05).

SPI-Ther again showed the highest hydroxyl radical scavenging activity (70.51%, Figure 11). SPI-Alk ranked second, whereas SPI-Try showed the lowest value. Together with the ABTS and DPPH results, this pattern suggests that thermolysin generated the strongest in vitro antioxidant response among the hydrolysates under the tested conditions. Because no peptide identification or direct mechanistic validation was performed, the contributions of specific amino acids or peptide sequences remain tentative [35,49]. This pattern is broadly consistent with previous studies showing that the hydroxyl radical scavenging activity of SPI hydrolysates depends strongly on protease selection and the resulting peptide profile [19,49]. Moreover, these chemical radical-scavenging assays are useful in vitro screening tools but do not necessarily predict antioxidant performance in vivo or in complex food systems.

### 3.11. Electronic Tongue Analysis

**Figure 12:**
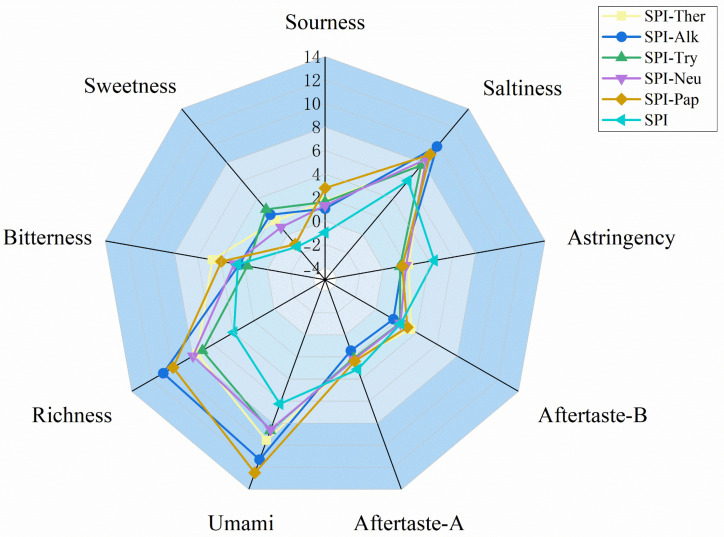
Radar chart of electronic tongue taste profiles of SPI and its enzymatic hydrolysates.

As showed as Figure 12, electronic tongue analysis showed that enzymatic hydrolysis reshaped the taste profile of SPI. Relative to native SPI, all hydrolysates exhibited enhanced umami and richness together with reduced astringency and aftertaste-A, indicating an overall shift toward a stronger savory profile after proteolysis. Among the different treatments, SPI-Ther showed the largest shift in the instrumental taste profile. The overall shift toward stronger umami/richness and reduced astringency is broadly consistent with previous reports showing that enzymatic treatment can markedly reshape the taste profile of soybean-derived protein hydrolysates. However, because published studies differ in substrate type, DH, and taste evaluation approach, the exact taste ranking among proteases is not expected to be identical across studies [51]. Although its bitterness (4.75) and aftertaste-B (3.46) were the highest, it also showed markedly elevated umami (9.57) and richness (7.57), suggesting that thermolysin generated a hydrolysate with stronger instrumental taste responses rather than a simply debittered product. The coexistence of stronger umami/richness with higher bitterness in SPI-Ther also agrees with earlier observations that enhanced functionality or stronger taste activity does not necessarily translate into debittering, but may instead reflect a functionality–taste trade-off [13]. Recent studies on SPI hydrolysates likewise indicate that enzymatic treatment can improve functional performance while simultaneously modulating bitterness, highlighting a functionality–taste trade-off rather than uniform sensory improvement [52]. In addition, recent work on SPI hydrolysates has emphasized that bitterness evaluation is best supported by direct sensory or e-tongue-related assessment, because bitterness cannot be inferred reliably from peptide composition alone [13]. This interpretation is consistent with the broader findings of the present study, in which thermolysin induced substantial structural remodeling, produced hydrolysates mainly below 25 kDa, released the highest level of free amino acids—especially Tyr and Phe—and delivered the strongest improvements in solubility, emulsifying activity, and antioxidant capacity. In contrast, SPI-Pap showed the highest umami score (12.49), SPI-Alk combined high saltiness (9.81) and richness (10.90) with relatively low bitterness (2.43), and SPI-Try exhibited the mildest overall taste profile, with the highest sweetness (2.82) and the lowest bitterness (1.72). These differences are useful for comparing relative taste tendencies among the hydrolysates in the present experimental system, but the electronic tongue results should be interpreted as instrument-based indications rather than as a substitute for human sensory validation. Therefore, under the tested conditions, thermolysin appeared to be the most favorable treatment for improving the measured functional attributes of SPI hydrolysates, although additional optimization or complementary debittering strategies would still be required when sensory mildness is a primary target. Likewise, the apparent umami advantage of papain and the milder taste profile of trypsin should be regarded as electronic tongue-based indications of relative taste tendency only.

### 3.12. Limitations of the Present Study

The present work should be interpreted as a comparative, condition-specific assessment of SPI hydrolysis under enzyme-specific practical conditions rather than as a comprehensive mechanistic analysis of intrinsic protease specificity. First, each protease was evaluated under its own literature-supported pH and temperature conditions, and the observed differences therefore reflect the combined effects of protease type and reaction conditions. Second, CD spectroscopy, free amino acid analysis, and electronic tongue measurements were performed on pooled samples, so these data mainly reflect analytical repeatability rather than variation among independently prepared hydrolysate batches. Third, no degree-of-hydrolysis determination, densitometric gel analysis, chromatographic peptide-size profiling, peptide identification, or human sensory validation was included. Accordingly, the present results support comparative structure–function–instrumental taste relationships under the tested conditions, while more detailed mechanistic interpretation requires further study.

## 4. Conclusions

Under the enzyme-specific hydrolysis conditions applied in this study, protease treatment and reaction environment jointly shaped the structural evolution, functional performance, and instrumental taste profile of SPI hydrolysates. Among the enzymes tested, thermolysin generated hydrolysates mainly below 25 kDa, induced marked conformational rearrangement, and released the highest level of free amino acids, especially tyrosine and phenylalanine. Under the present conditions, thermolysin also produced the highest solubility, the highest emulsifying activity index, and the strongest ABTS, DPPH, and hydroxyl radical scavenging activities, indicating that it was the most effective enzyme for functional intensification of SPI in this study. However, this advantage was accompanied by a clear sensory trade-off. Electronic tongue analysis showed that SPI-Ther exhibited enhanced umami and richness, but also the highest bitterness and aftertaste-B, indicating that thermolysin intensified overall taste expression rather than providing direct debittering under the present hydrolysis conditions. By comparison, papain produced the strongest umami response, whereas trypsin generated the mildest taste profile and the lowest bitterness. Therefore, thermolysin should be regarded primarily as a treatment for functional enhancement of SPI under the present enzyme-specific hydrolysis conditions. These conclusions should be interpreted as condition-specific comparative findings rather than a comprehensive mechanistic analysis, because the present study did not include unified reaction conditions across proteases, degree-of-hydrolysis determination, peptide profiling, or human sensory validation. Future work should more explicitly address the two main missing links between the present structural/functional results and the interpretation of taste behavior, namely peptide characterization and sensory validation. Specifically, further studies should include peptide identification and molecular-weight profiling to link specific hydrolysis products with the observed functional and taste responses, together with trained sensory-panel or consumer validation to confirm the instrumental taste trends before taste-oriented formulations are recommended.

## Figures and Tables

**Table 1:** Preparation of samples for SDS-PAGE analysis.

Sample	Concentration (μg/μL)	Sample Vol. (μL)	H_2_O (μL)	4× LDS Buffer (μL)	Total Vol. (μL)
SPI	3.43	8.75	6.25	5.0	20
SPI-Ther	2.54	11.81	3.19	5.0	20
SPI-Alk	6.30	4.76	10.24	5.0	20
SPI-Pap	6.55	4.58	10.42	5.0	20
SPI-Neu	5.79	5.18	9.82	5.0	20
SPI-Try	5.62	5.34	9.66	5.0	20

**Table 2:** Secondary structure composition of soy protein isolate (SPI) and its enzymatic hydrolysates.

Sample	α-Helix	β-Sheet	β-Turn	Random Coil
SPI	0.131 ± 0.009 ^a^	0.199 ± 0.007 ^b^	0.130 ± 0.012 ^a^	0.540 ± 0.022 ^a^
SPI-Ther	0.013 ± 0.001 ^e^	0.343 ± 0.012 ^a^	0.139 ± 0.017 ^a^	0.505 ± 0.019 ^ab^
SPI-Alk	0.019 ± 0.003 ^de^	0.339 ± 0.015 ^a^	0.138 ± 0.014 ^a^	0.504 ± 0.012 ^ab^
SPI-Pap	0.026 ± 0.002 ^d^	0.332 ± 0.012 ^a^	0.139 ± 0.008 ^a^	0.503 ± 0.014 ^ab^
SPI-Try	0.058 ± 0.003 ^b^	0.325 ± 0.011 ^a^	0.128 ± 0.007 ^a^	0.489 ± 0.011 ^b^
SPI-Neu	0.050 ± 0.004 ^c^	0.328 ± 0.010 ^a^	0.131 ± 0.012 ^a^	0.491 ± 0.010 ^b^

Note: Values are mean ± SD of three parallel measurements performed on a composite sample prepared by pooling three independently prepared hydrolysate batches for each treatment. Different superscript letters within the same column indicate significant differences (*p* < 0.05).

**Table 3:** Amino acid composition and content of soy protein isolate (SPI) hydrolysates prepared by different proteases.

Classification	Amino Acid	SPI-Ther (mg/g)	SPI-Alk (mg/g)	SPI-Pap (mg/g)	SPI-Try (mg/g)	SPI-Neu (mg/g)
Hydrophobic	Ala	0.105 ± 0.002 ^bc^	0.128 ± 0.003 ^b^	0.139 ± 0.003 ^ab^	0.148 ± 0.003 ^a^	0.068 ± 0.002 ^d^
	Val	0.189 ± 0.003 ^c^	0.194 ± 0.004 ^c^	0.346 ± 0.006 ^a^	0.175 ± 0.003 ^c^	0.134 ± 0.003 ^d^
	Met	0.131 ± 0.003 ^a^	0.064 ± 0.002 ^c^	0.115 ± 0.002 ^b^	0.115 ± 0.003 ^b^	0.119 ± 0.003 ^b^
	Ile	0.155 ± 0.003 ^d^	0.279 ± 0.004 ^c^	0.245 ± 0.005 ^c^	0.349 ± 0.006 ^ab^	0.353 ± 0.007 ^a^
	Leu	0.122 ± 0.003 ^b^	0.026 ± 0.001 ^d^	0.084 ± 0.002 ^c^	0.161 ± 0.003 ^a^	0.134 ± 0.003 ^b^
	Tyr	4.928 ± 0.043 ^a^	3.188 ± 0.038 ^c^	3.459 ± 0.044 ^b^	0.778 ± 0.013 ^d^	0.619 ± 0.009 ^e^
	Phe	3.355 ± 0.047 ^a^	0.674 ± 0.011 ^b^	0.348 ± 0.006 ^c^	0.364 ± 0.006 ^c^	0.254 ± 0.004 ^d^
	Pro	0.370 ± 0.010 ^c^	0.532 ± 0.010 ^b^	0.839 ± 0.010 ^a^	0.559 ± 0.015 ^b^	0.290 ± 0.010 ^d^
Basic	His	0.411 ± 0.006 ^a^	0.106 ± 0.002 ^c^	0.192 ± 0.003 ^b^	0.101 ± 0.002 ^c^	0.093 ± 0.002 ^c^
	Lys	0.412 ± 0.006 ^b^	0.223 ± 0.003 ^c^	0.223 ± 0.003 ^c^	0.470 ± 0.010 ^a^	0.055 ± 0.001 ^d^
	Arg	1.282 ± 0.013 ^b^	0.837 ± 0.007 ^c^	1.240 ± 0.020 ^b^	2.888 ± 0.022 ^a^	0.568 ± 0.004 ^d^
Hydrophilic	Asp	0.099 ± 0.003 ^c^	0.098 ± 0.002 ^c^	0.219 ± 0.004 ^a^	0.233 ± 0.004 ^a^	0.180 ± 0.003 ^b^
	Thr	0.113 ± 0.002 ^b^	0.058 ± 0.001 ^d^	0.047 ± 0.001 ^e^	0.152 ± 0.003 ^a^	0.066 ± 0.001 ^c^
	Ser	0.123 ± 0.002 ^c^	0.105 ± 0.001 ^d^	0.204 ± 0.004 ^a^	0.169 ± 0.003 ^b^	0.072 ± 0.001 ^e^
	Glu	0.117 ± 0.002 ^c^	0.214 ± 0.004 ^ab^	0.229 ± 0.004 ^a^	0.203 ± 0.003 ^b^	0.087 ± 0.002 ^d^
	Gly	0.092 ± 0.002 ^c^	0.098 ± 0.002 ^c^	0.092 ± 0.002 ^c^	0.141 ± 0.003 ^a^	0.088 ± 0.002 ^c^
Sulfur-containing	Cys	2.801 ± 0.024 ^a^	2.666 ± 0.022 ^b^	2.644 ± 0.022 ^b^	2.536 ± 0.019 ^c^	2.327 ± 0.018 ^d^
Total	--	14.805 ± 0.125 ^a^	9.490 ± 0.082 ^d^	10.665 ± 0.095 ^b^	9.536 ± 0.078 ^cd^	5.507 ± 0.046 ^e^

Note: Values are mean ± SD of three parallel injections performed on a composite sample prepared by pooling three independently prepared hydrolysate batches for each treatment. These data reflect analytical repeatability of the pooled sample rather than variation among independent hydrolysate preparations. Different superscript letters within the same column indicate significant differences (*p* < 0.05).

## Data Availability

The original contributions presented in this study are included in the article. Further inquiries can be directed to the corresponding author.
